# Perioperative medicine – a vital but neglected part of success in surgery

**DOI:** 10.1515/iss-2019-2036

**Published:** 2019-12-24

**Authors:** Wolfgang Schwenk, Stephan M. Freys

**Affiliations:** Professor of Surgery, Chairman, Department of General-, Visceral- and Vascular Surgery, Städtisches Klinikum Solingen gGmbH, Gotenstrasse 1, D-42653 Solingen, Germany; Department of Surgery, DIAKO Evangelisches Diakonie-Krankenhaus gGmbH, Bremen, Gemany

This issue of *Innovative Surgical Sciences* (*ISS*) is dedicated to the perioperative treatment of surgical patients. A topic that has been neglected by many surgeons for far too long. While new surgical techniques are quickly adopted, implementing modern perioperative therapies seems to be much more difficult. The introduction of minimally invasive surgery (MIS) is a good example of the rapid spread of a new operative technique. Despite the early lack of high-level evidence [1] MIS was introduced for many abdominal operations within a few years after the initial reports of successful laparoscopic cholecystectomy. More recently, a great desire in the surgical community can be observed to implement robotic surgery into the clinical routine although there is no high-level evidence from randomized controlled trials that robotic surgery will have any benefit for the patient. While the benefits of MIS are proven beyond any doubt today, we will have to see whether the same will be true for robotics in the future.

In contrast to these examples optimized perioperative treatment is being adopted by surgeons very slowly. Twenty-five years ago Henrik Kehlet and colleagues published their initial case series of eight elderly patients undergoing resection of colonic cancer in a fast-track regimen with a postoperative hospital stay of only 2 days [2]. Since then numerous clinical trials and randomized controlled studies have demonstrated the major benefits for patients undergoing surgery with optimized perioperative treatment: enhanced recovery, lesser morbidity, improved mobilization, shortened time to normal daily activities, and massively reduced the duration of postoperative hospital stay [3]. These results of fast-track- or enhanced recovery after surgery (ERAS)-regimens were shown in open as well as MIS [4] and a vast variety of operations in general, abdominal [5], gynaecological [6], urological [7] and orthopedic or trauma surgery [8].

In this issue of *ISS* some of the cornerstones of optimal perioperative medicine are highlighted: perioperative nursing care, perioperative nutrition, acute pain medicine and rational treatment of patients under anticoagulation therapy. Furthermore, a thorough review of the past, present, and future of perioperative medicine is provided. We do hope that readers of *ISS* will adopt the strategies given in these articles to optimize the perioperative treatment of their patients. Optimal results of surgery will always be based on a perfect surgical technique. However, striving for optimal technique alone while neglecting optimal perioperative therapy will never achieve the best outcome possible for our patients. We have to embrace and implement modern perioperative medicine because surgery is so much more than just operating the patient!

## References

[1] Horton R Surgical research or comic opera: questions, but few answers. Lancet 1996;347:984–5. 8606606 10.1016/s0140-6736(96)90137-3

[2] Bardram L , Funch-Jensen P , Jensen P , Crawford ME , Kehlet H Recovery after laparoscopic colonic surgery with epidural analgesia, and early oral nutrition and mobilisation. Lancet 1995;345:763–4. 7891489 10.1016/s0140-6736(95)90643-6

[3] Greer NL , Gunnar WP , Dahm P , Lee AE , MacDonald R , Shaukat A , Enhanced recovery protocols for adults undergoing colorectal surgery: a systematic review and meta-analysis. Dis Colon Rectum 2018;61:1108–18. 30086061 10.1097/DCR.0000000000001160

[4] Li Z , Zhao Q , Bai B , Ji G , Liu Y Enhanced recovery after surgery programs for laparoscopic abdominal surgery: a systematic review and meta-analysis. World J Surg 2018;42:3463–73. 29750324 10.1007/s00268-018-4656-0

[5] Visioni A , Shah R , Gabriel E , Attwood K , Kukar M , Nurkin S Enhanced recovery after surgery for noncolorectal surgery?: a systematic review and meta-analysis of major abdominal surgery. Ann Surg 2018;267:57–65. 28437313 10.1097/SLA.0000000000002267

[6] Trowbridge ER , Dreisbach CN , Sarosiek BM , Dunbar CP , Evans SL , Hahn LA , Review of enhanced recovery programs in benign gynecologic surgery. Int Urogynecol J 2018;29:3–11. 28871417 10.1007/s00192-017-3442-0

[7] Di Rollo D , Mohammed A , Rawlinson A , Douglas-Moore J , Beatty J Enhanced recovery protocols in urological surgery: a systematic review. Can J Urol 2015;22:7817–23. 26068632

[8] Zhu S , Qian W , Jiang C , Ye C , Chen X Enhanced recovery after surgery for hip and knee arthroplasty: a systematic review and meta-analysis. Postgrad Med J 2017;93:736–42. 28751437 10.1136/postgradmedj-2017-134991 PMC5740550

